# The pattern of TSH and fT4 levels across different BMI ranges in a large cohort of euthyroid patients with obesity

**DOI:** 10.3389/fendo.2022.1029376

**Published:** 2022-10-13

**Authors:** Chiara Mele, Stefania Mai, Tiziana Cena, Loredana Pagano, Massimo Scacchi, Bernadette Biondi, Gianluca Aimaretti, Paolo Marzullo

**Affiliations:** ^1^ Department of Translational Medicine, University of Piemonte Orientale, Novara, Italy; ^2^ Istituto Auxologico Italiano, IRCCS, Laboratory of Metabolic Research, S. Giuseppe Hospital, Piancavallo, Italy; ^3^ Division of Endocrinology, Diabetology and Metabolism, Department of Medical Sciences, University of Turin, Turin, Italy; ^4^ Istituto Auxologico Italiano, IRCCS, Division of General Medicine, S. Giuseppe Hospital, Piancavallo, Italy; ^5^ Department of Clinical Sciences and Community Health, University of Milan, Milan, Italy; ^6^ Department of Clinical Medicine and Surgery, University of Naples Federico II, Naples, Italy

**Keywords:** obesity, thyroid, metabolism, body composition, leptin

## Abstract

**Purpose:**

A multifold association relates the hypothalamo-pituitary-thyroid axis to body weight. The potential underlying mechanisms are incompletely understood. Further, the mild severity of obesity and the small proportion of individuals with obesity in so far published cohort studies provide little insights on metabolic correlates of thyroid function in obesity.

**Methods:**

We retrospectively enrolled 5009 adults with obesity (F/M, 3448/1561; age range, 18-87 years; BMI range, 30.0-82.7 kg/m^2^), without known thyroid disease in a study on TSH and fT4 levels, lipid profile, glucose homeostasis and insulin resistance, anthropometric parameters including BIA-derived fat mass (%FM) and fat-free mass (FFM).

**Results:**

The overall reference interval for TSH in our obese cohort was 0.58-5.07 mIU/L. As subgroups, females and non-smokers showed higher TSH levels as compared to their counterparts (p<0.0001 for both), while fT4 values were comparable between groups. There was a significant upward trend for TSH levels across incremental BMI classes in females, while the opposite trend was seen for fT4 levels in males (p<0.0001 for both). Expectedly, TSH was associated with %FM and FFM (p<0,0001 for both). TSH and fT4 showed correlations with several metabolic variables, and both declined with aging (TSH, p<0.0001; fT4, p<0.01). In a subgroup undergoing leptin measurement, leptin levels were positively associated with TSH levels (p<0.01). At the multivariable regression analysis, in the group as a whole, smoking habit emerged as the main independent predictor of TSH (β=-0.24, p<0.0001) and fT4 (β=-0.25, p<0.0001) levels. In non-smokers, %FM (β=0.08, p<0.0001) and age (β=-0.05, p<0.001) were the main significant predictors of TSH levels. In the subset of nonsmokers having leptin measured, leptin emerged as the strongest predictor of TSH levels (β=0.17, p<0.01).

**Conclusions:**

Our study provides evidence of a gender- and smoking-dependent regulation of TSH levels in obesity.

## Introduction

The relationship between obesity and thyroid function is complex and bidirectional. Thyroid dysfunctions can influence changes in body weight due to the well-known control exerted by thyroid hormones on thermogenesis and calorie ingestion (1, 2). In the last few decades, however, growing evidence accumulated on the ability of obesity to promote per se changes in circulating markers of thyroid function (3–14). Cross-sectional and longitudinal studies have shown that increasing bodyweight associates with subsequent increments in TSH within the normal range (3–14), with accumulation of visceral adipose tissue rather than metabolic impairment driving this association (8, 15, 16). It has been suggested that up to 25% of subjects with obesity can harbor moderately elevated TSH concentrations, usually below the threshold of 10 µIU/mL, devoid of underlying thyroid disease (13, 17). With regards to thyroid hormones, in obesity free triiodothyronine (fT3) levels tend to increase (17–19), while fT4 levels tend to decline with increasing body weight (13, 20, 21). Further, changes in thyroid function, even within the normal range, have been claimed to contribute per se to the onset of the metabolic syndrome, such that an association has been reported between circulating TSH and visceral obesity, dyslipidemia, insulin resistance, hypertension and non-alcoholic fatty liver disease (NAFLD) (22–25).

The process underlying this interaction is complex and involves the influence of adipose tissue on the hypothalamus-pituitary-thyroid (HPT) axis through a loop that is mediated by leptin and is critical for thyroid modulation of energy homeostasis (26). Leptin controls the TRH gene promoter in the hypothalamic paraventricular nucleus (27, 28) and acts directly and indirectly to increase the production of α-melanocyte stimulating hormone (α-MSH), the ligand of the melanocortin receptor-4 (MC4R) (29). Thus, in condition of positive energy balance, leptin intervenes to upregulate the HPT axis and stimulate the central activation of the melanocortin pathway to promote energy expenditure (30, 31). In humans, TSH has been repeatedly shown to correlate with circulating leptin (6, 12), with smoking playing a potential role in this association (32).

So far, cohort studies have included limited proportions of obese persons and with moderate degree of obesity, thus it remains difficult to draw definitive conclusions on global correlates of thyroid function parameters in all degrees of obesity. Further, lack of adjustment for important covariates limits the strength of the reported associations between thyroid and adiposity parameters (4, 11–13, 18, 33, 34).

The current retrospective study was primarily designed to investigate the pattern of TSH and FT4 levels across different BMI ranges in a large cohort of euthyroid patients with obesity. Secondary aims of the study were to explore the metabolic correlates of thyroid function, as well as the influence of covariates on thyroid function parameters. In a subgroup, we lastly aimed to assess the role of serum leptin on circulating TSH and FT4 levels.

## Patients and methods

### Patients

Of 6412 consecutive *de novo* patients admitted between 7 January 2014 and 30 September 2018 to our institution for workup and rehabilitation of obesity, 5009 euthyroid adults were included in the study (3448 females/1561 males; age, 50.0 ± 15.2 years; body mass index (BMI), 44.3 ± 7.4 kg/m^2^). Exclusion criteria were the presence of previously known or unknown thyroid dysfunction or Hashimoto’s thyroiditis; any morbidity potentially affecting thyroid function parameters including acute illnesses and/or acute inflammation and/or chronic conditions; any endocrine disorders; use of levothyroxine (LT4) or triiodothyronine (T3) or medications potentially interfering with thyroid function (such as amiodarone, steroids, or lithium carbonate therapy); pregnancy. In all patients, body weight was stable at least for three months or longer prior to study enrolment.

The investigation was approved by the local ethics committee, functioning according to the fourth edition of the Guidelines on the Practice of Ethics Committees in Medical Research With Human Participants on admission, and written consent was obtained from all patients.

### Body measurements

Body measurements were conducted on fasting patients wearing light underwear. Weight and height were measured to the nearest 0.1 kg and 0.1 cm, respectively, and BMI was expressed as body mass (kg)/height (m^2^). The criterion for obesity was BMI ≥ 30 kg/m^2^.

Waist circumference (WC) was measured midway between the lowest rib and the top of the iliac crest after gentle expiration.

Bioimpedance analysis (BIA, 101/S Akern; Florence, Italy) allowed measurement of percent fat mass (%FM) and fat-free mass (FFM, kg). Methodology, variation coefficients, and exclusion criteria have been detailed previously (35).

### Laboratory tests

Undiluted serum samples were assayed in duplicate for fT4 and TSH using an automated chemiluminescence assay system (Immulite 2000; DPC, Los Angeles, CA). The principle of the method is a two-site, solid-phase chemiluminescent immunometric assay or competitive immunoassay. Normal values for TSH are 0.27–4.20 mIU/L, and for fT4 9.0-17.0 pg/mL.

Routine laboratory data included glucose, total cholesterol (CHO), high-density (HDL) and low-density lipoprotein (LDL) cholesterol and triglycerides (TG) measured by enzymatic methods (Roche Diagnostics, Mannheim, Germany).

Insulin levels were measured using a Cobas Integra 800 Autoanalyzer (Roche Diagnostics, Indianapolis, IN, USA), and insulin resistance was expressed as homeostatic model assessment of insulin resistance (HOMA-IR) [insulin (mIU/L) x glucose (mmol/L)/22.5]. HOMA-IR cutoff for insulin resistance was 2.5 (36). Type 2 diabetes mellitus (T2DM) was ascertained by patients’ history and/or biochemistry analyses on admission according to current guidelines (37).

In a subgroup of 502 patients, we measured serum leptin levels by commercial Linco RIA kit (Linco, St. Louis, MO) having detection limit of 0.15 μg/liter, intraassay coefficients of variation (CVs) of 2.2% at 6 μg/liter, 2.7% at 25 μg/liter, and 5.9% at 62.8 μg/liter and interassay CVs of 4.3, 4, and 6.9% at the concentration of 5.1, 21, and 56.2 μg/liter, respectively.

### Statistical analysis

Statistical analysis was performed using SPSS version 21 (Somers, NY, USA). Values are expressed as means ± standard deviation (SD) or percentage. Data points not normally distributed obtained by the Shapiro–Wilk test were log-transformed to improve the symmetry and homoscedasticity of the distribution. For comparative analysis, univariate ANOVA between groups were used. Repeated Measures ANOVA was used to evaluate comparison of TSH and fT4 levels within BMI stratification classes, and Hyun-Feldt correction was applied when the assumption of sphericity, tested using Mauchly’s test, was violated. Pearson’s correlation analysis and the Chi square were used to identify significant associations between variables of interest. Stepwise multivariable regression analysis was used to evaluate the independent association of TSH or fT4 levels with metabolic, anthropometric or biochemical parameters. The multilinear model included several combinations of independent variables encompassing age, gender, smoking habit, BMI, WC, WHR, lipid panel, body composition and leptin levels. β coefficients and related significance values obtained from the models are reported. P < 0.05 was considered as statistically significant.

## Results

### Clinical and biochemical characteristics of patients

**Figure 1:**
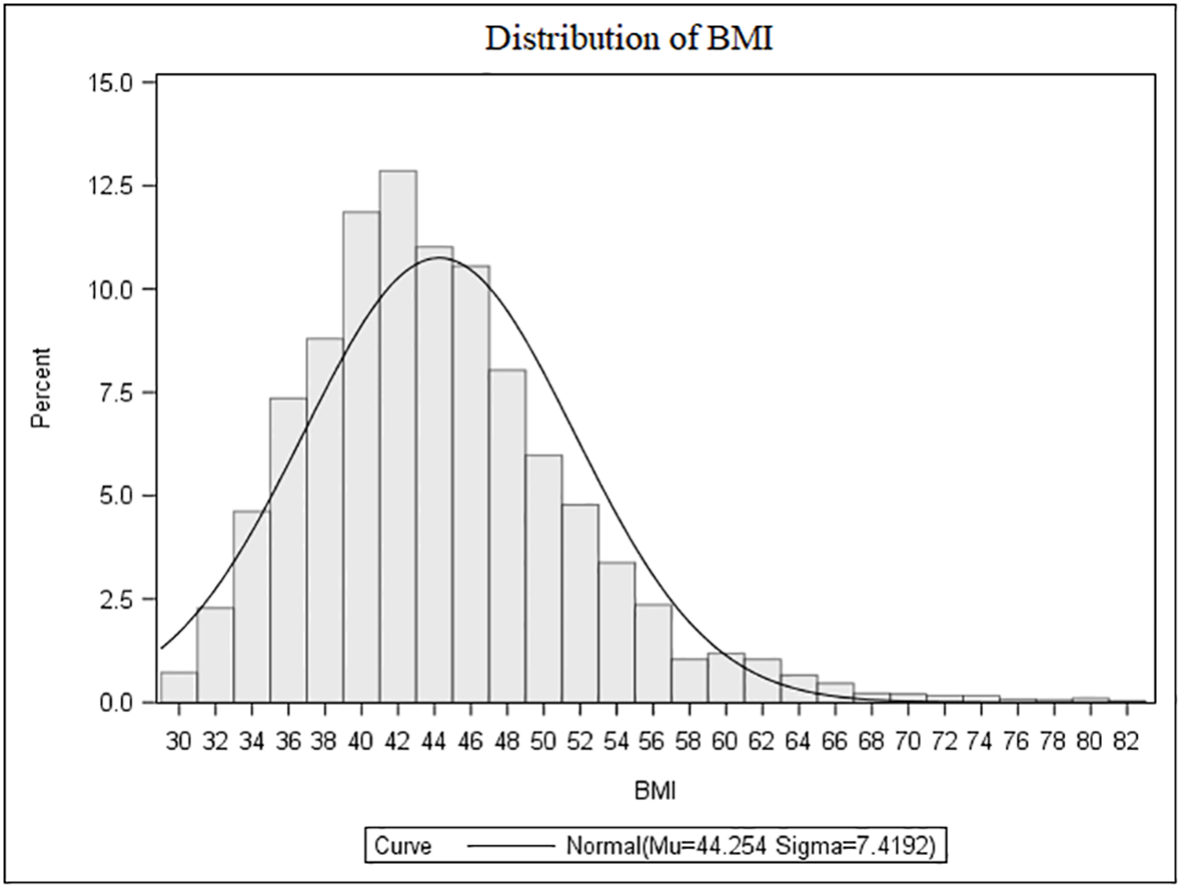
Distribution of BMI values in the whole population.

The population characteristics are summarized in **Table 1**. Females accounted for 68.8% of the cohort (male-to-female ratio, 1:2.2). Overall, BMI was ≥40.0 kg/m^2^ in 71.3%, >35.0 to 39.9 kg/m2 in 21.1%, >30.0 to 34.9 kg/m2 in 7.6% of cases. Among those classified as severely obese (≥40.0 kg/m2), 25.9% had a BMI ≥50.0 kg/m^2^ (**Figure 1**).

**Table 1:** Summary of anthropometric and metabolic variables in the population as a whole and subgrouped according to gender and smoking habit.

Parameters	Whole population	Females	Males	Smokers	Non-smokers
Gender (F/M, N, %)	3448/1561	**68.8%**	**31.2%^b^**	**729/414**	**2719/1147^f^**
Age (years)	50.0 ± 15.2	**50.8 ± 15.6**	**48.2 ± 14.3^b^**	50.2 ± 13.7	50.0 ± 15.6
Smoking habit	1143 (22.8%)	**729 (21.1%)**	**414 (26.5%)^b^**	–	–
BMI (kg/m^2^)	44.3 ± 7.4	**44.0 ± 7.3**	**44.7 ± 7.7^a^**	44.3 ± 7.5	44.2 ± 7.4
BMI ≥ 40 kg/m^2^ (N, %)	3569 (71.3%)	2430 (70.5%)	1139 (73.0%)	818 (71.6%)	2751 (71.2%)
WC (cm)	124.3 ± 16.5	**119.3 ± 14.5**	**135.1 ± 15.3^b^**	**125.2 ± 16.3**	**124.0 ± 16.5^c^**
TSH (mIU/L)	2.08 ± 1.20	**2.16 ± 1.27**	**1.92 ± 1.01^b^**	**1.54 ± 0.80**	**2.25 ± 1.25^f^**
fT4 (pg/mL)	11.54 ± 1.73	11.56 ± 1.72	11.56 ± 1.74	**10.75 ± 1.48**	**11.77 ± 1.73^f^**
Glucose (mg/dL)	103.4 ± 33.5	**101.5 ± 32.5**	**107.5 ± 35.2^b^**	104.1 ± 34.0	103.2 ± 33.4
Insulin (mIU/L)	15.9 ± 9.9	**14.9 ± 9.4**	**17.9 ± 10.6^b^**	15.7 ± 8.7	15.9 ± 10.2
HOMA-IR	4.2 ± 3.4	**3.8 ± 3.2**	**4.8 ± 3.7^b^**	4.1 ± 2.9	4.2 ± 3.6
CHO (mg/dL)	200.9 ± 41.7	**203.1 ± 41.4**	**196.1 ± 42.0^b^**	200.5 ± 40.8	201.1 ± 41.9
LDL-CHO (mg/dL)	125.9 ± 35.4	**127.3 ± 35.2**	**122.7 ± 35.7^b^**	125.9 ± 35.2	125.8 ± 35.5
HDL-CHO (mg/dL)	45.3 ± 12.3	**48.2 ± 12.4**	**38.9 ± 9.3^b^**	**44.0 ± 11.5**	**45.7 ± 12.5^f^**
TG (mg/dL)	149.2 ± 74.4	**138.6 ± 65.8**	**172.8 ± 86.0^b^**	**153.6 ± 76.0**	**147.9 ± 73.8^c^**
Leptin (μg/l)*	40.3 ± 21.0	**45.7 ± 20.5**	**25.3 ± 14.1^b^**	40.0 ± 19.2	40.4 ± 21.6
FM (%)	46.7 ± 6.9	**49.2 ± 5.4**	**41.1 ± 6.6^b^**	**46.2 ± 7.0**	**46.9 ± 6.9^d^**
FFM (kg)	62.2 ± 14.8	**51.0 ± 5.5**	**59.1 ± 6.6^b^**	**54.0 ± 7.0**	**53.3 ± 6.9^d^**

Data are expressed as mean ± SD, absolute number and percentage. Comparison between groups was performed by using univariate ANOVA and χ2 test. Significant differences are shown in bold characters.

For significance: females vs males, ^a^p<0.01, ^b^p<0.0001; smokers vs non-smokers, ^c^p<0.05, ^d^p<0.01, p<0.001, ^f^p<0.0001.

BMI, body mass index; CHO, cholesterol; FM, fat mass; FFM, fat-free mass; fT4, free thyroxine; HOMA-IR, Homeostatic Model of Insulin Resistance; TG, triglycerides; TSH, thyroid-stimulating hormone; WC, waist circumference.

*Leptin was measured in 502 patients.

**Figure 2:**
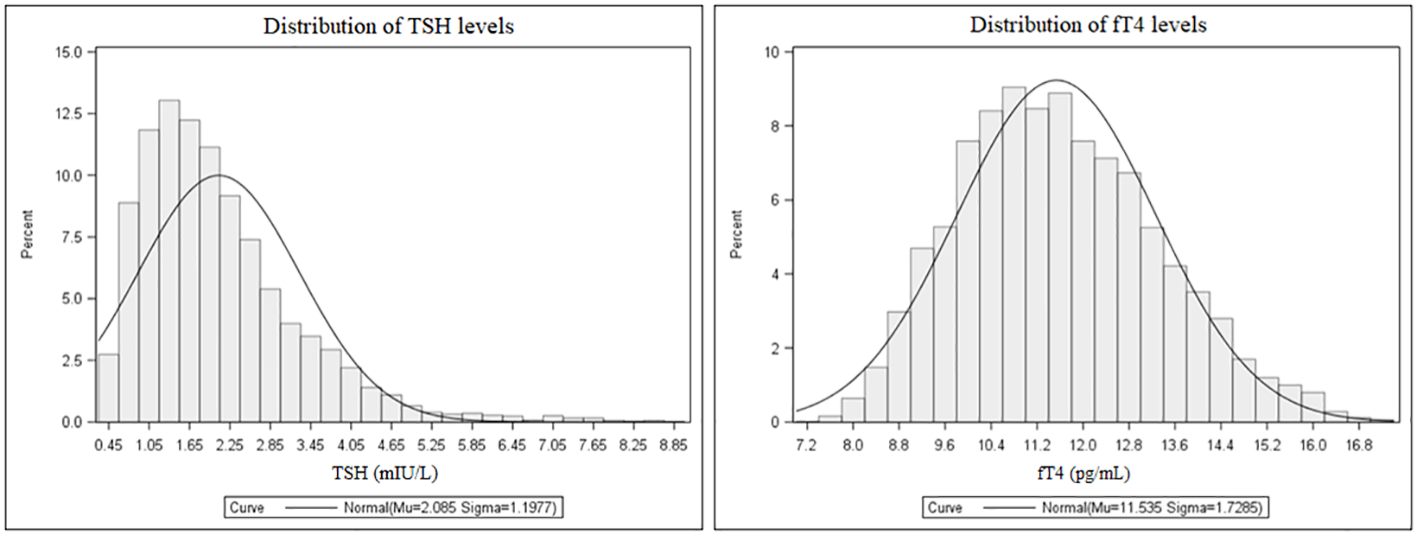
Distribution of TSH (left) and fT4 levels (right) in the whole population.

The distribution of TSH and fT4 levels in the entire population are shown in **Figure 2**. Mean TSH and fT4 levels were 2.08 ± 1.20 mIU/L and 11.54 ± 1.73 pg/mL, respectively. The 2.5th-97.5th percentile interval for TSH was 0.58-5.07 mIU/L, and 276 subjects (5.5%) had their TSH above the upper limit of the normal immunoassay range. In this subset, TSH values were 5.4 ± 1.1 mIU/L. In 264 subjects (5.3%), fT4 values were below the normal range (mean ± SD, 8.5 ± 0.4 pg/mL). All patients with low fT4 had their TSH in the normal immunoassay range and viceversa, and anti-thyroid antibodies were negative in all patients with altered TSH and fT4 concentrations. There were, as expected, metabolic differences according to gender as well as smoking habit. As subgroups, females had higher TSH levels than males (p<0.0001), and smokers had lower TSH and fT4 levels compared to their counterpart (p<0.0001 for both).

**Figure 3:**
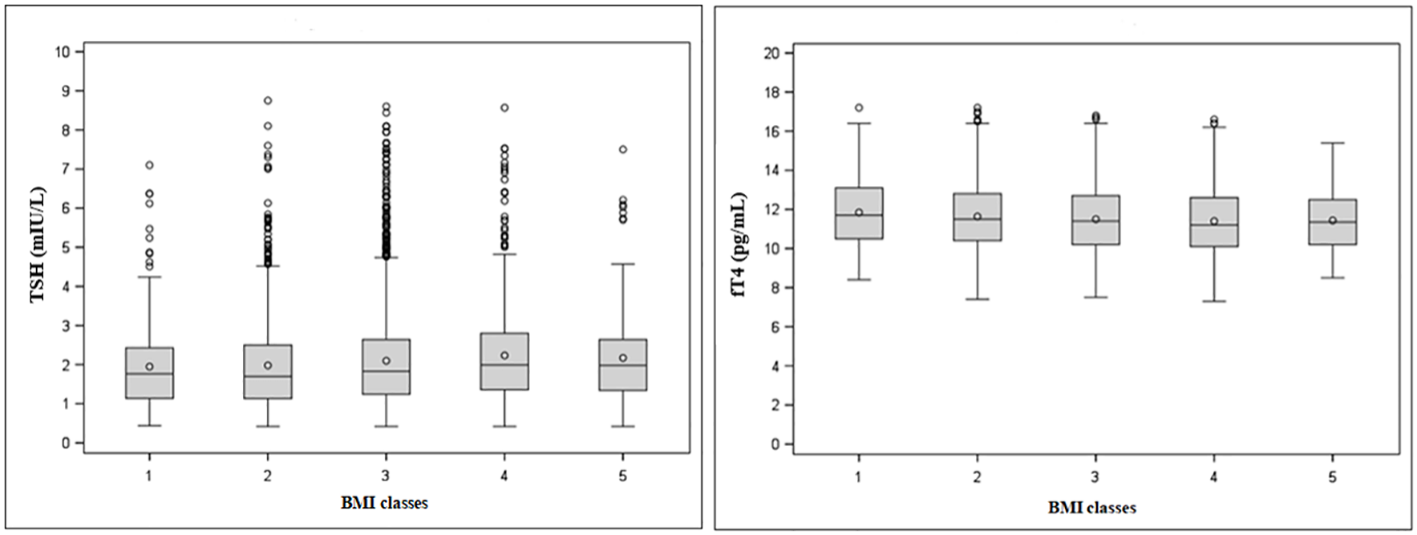
Tukey’s box-and-whisker plot of TSH (left) and fT4 levels (right) according to BMI classes. Outliers are plotted as individual points.

**Figure 4:**
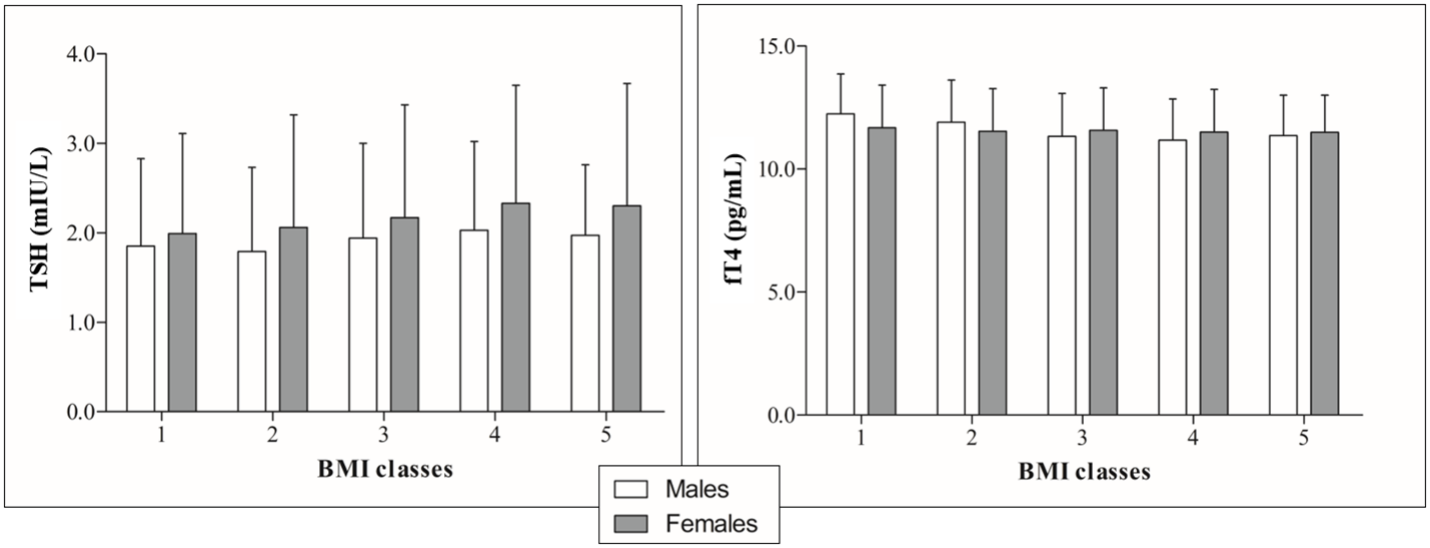
TSH (right) and fT4 profiles (left) by incremental BMI classes in the population subgrouped according to gender.

Stratification by BMI (**Table 2** and **Figure 3**) revealed a significant upward trend for TSH (F=6.46, p<0.0001) and a significant downward trend for fT4 levels across five incremental BMI classes (F=5.53, p<0.0001). With BMI class 1 as reference, TSH increased by 0.03 mIU/L (p=0.43) in class 2, 0.15 mIU/L (p<0.05) in class 3, 0.29 mIU/L (p<0.001) in class 4, and 0.22 mIU/L (p<0.05) in class 5. In parallel, fT4 decreased by 0.21 pg/mL (p<0.05) in BMI class 2, 0.35 pg/mL (p<0.001) in class 3, 0.45 pg/mL (p<0.0001) in class 4, and 0.41 pg/mL (p<0.01) in class 5 compared to BMI class 1. In gender-based analysis (**Figure 4**), females exhibited a significant upward trend for TSH (F=5.09, p<0.0001), which approached statistical significance also in males (F=2.30, p=0.06). Contrariwise, a decrease in fT4 was only seen in males (F=13.50, p<0.0001) and not in females (F=0.62, p=0.65) across BMI classes.

**Table 2:** TSH and fT4 levels in the study population according to BMI stratified in 5 classes.

		N° of patients	TSH (mIU/L) (mean ± SD)	fT4 (pg/mL) (mean ± SD)
**BMI stratification classes**	**1**: BMI ≤ 34.9	366	1.95 ± 1.08	11.85 ± 1.72
	**2**: 34.9<BMI ≤ 39.9	1054	1.98 ± 1.18	11.64 ± 1.75
	**3**: 39.9<BMI ≤ 49.9	2646	2.10 ± 1.21	11.50 ± 1.73
	**4**: 49.9<BMI ≤ 59.9	753	2.23 ± 1.23	11.40 ± 1.72
	**5**: BMI>59.9	190	2.17 ± 1.18	11.44 ± 1.56
**p-value (repeated measures ANOVA)**			**<0.0001**	**<0.0001**

Data are expressed as mean ± SD. Comparison across BMI strata was performed by using repeated measures ANOVA with Hyun-Feldt correction as appropriate. Significant differences are shown in bold characters.

BMI, body mass index; fT4, free thyroxine; TSH, thyroid-stimulating hormone.

In restricting the analysis to patients with extreme obesity (BMI>50 kg/m^2^), which represented 18.4% of the entire population, TSH levels appeared to plateau as compared to the lower BMI class. In this BMI class, not only TSH levels differed between females and males (2.31 ± 1.33 vs. 2.01 ± 0.94 mIU/L, p<0.001), but the same occurred also for fT4 levels (11.51 ± 1.70 vs. 11.21 ± 1.65 pg/mL, p=0.02).

### Correlation and regression analyses

TSH and fT4 levels were tested in relation to demographic, anthropometric and metabolic parameters (**Table 3**). TSH but not fT4 levels were significantly correlated to body measures, particularly %FM and FFM. Both thyroid parameters were related to several metabolic variables, but the strongest association was seen in relation to the smoking habit. After controlling for BMI, age, gender and smoking habit, these associations remained all significant except for that relating TSH to insulin and HDL levels, and between fT4 and WC.

**Table 3:** Bivariate correlation analysis between serum TSH or fT4 levels and demographic, anthropometric, biochemical and metabolic variables.

Variables	TSH (mIU/L)		fT4 (pg/mL)	
		r	p	r	p
Age (years)	**-0.05**	**<0.0001**	**-0.04**	**0.002**
Smoking habit (No=0, Yes=1)	**-0.25**	**<0.0001**	**-0.25**	**<0.0001**
BMI (kg/m^2^)	**0.07**	**<0.0001**	**-0.05**	**<0.0001**
WC (cm)	-0.01	0.65	**-0.06**	**<0.0001**
Glucose (mg/dL)	**-0.05**	**0.001**	**0.03**	**0.04**
Insulin (mIU/L)	**0.03**	**0.04**	-0.02	0.09
HOMA-IR	-0.01	0.67	-0.01	0.67
CHO (mg/dL)	**0.04**	**0.006**	-0.02	0.20
LDL-CHO (mg/dL)	0.02	0.15	-0.02	0.21
HDL-CHO (mg/dL)	**0.04**	**0.007**	**0.07**	**<0.0001**
TG (mg/dL)	0.03	0.05	**-0.06**	**<0.0001**
Leptin (μg/l)*	**0.14**	**0.002**	0.01	0.91
FM (%)	**0.11**	**<0.0001**	0.001	0.93
FFM (kg)	**-0.11**	**<0.0001**	-0.001	0.94

Significant associations are shown in bold characters.

BMI, body mass index; CHO, cholesterol; FM, fat mass; FFM, fat-free mass; fT4, free thyroxine; HOMA-IR, Homeostatic Model of Insulin Resistance; REE, resting energy expenditure; TG, triglycerides; TSH, thyroid-stimulating hormone; WC, waist circumference.

*Leptin was measured in 502 patients.

**Figure 5:**
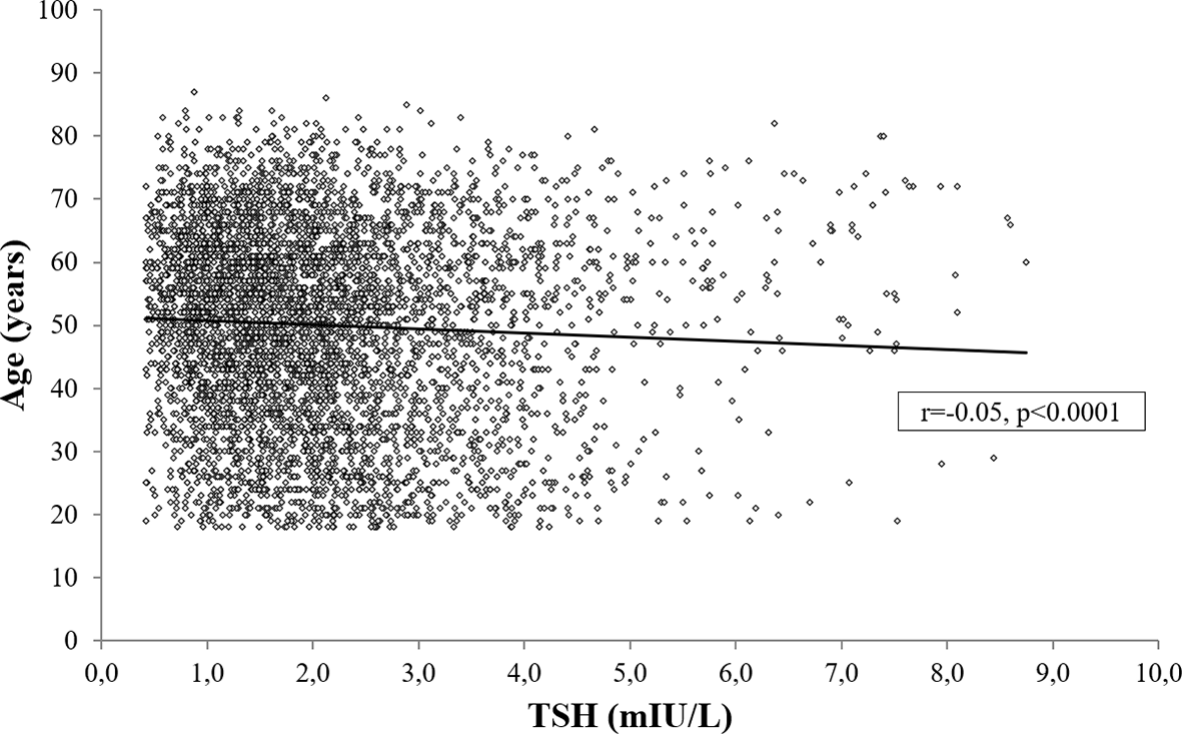
Bivariate correlation analysis between serum TSH and age.

Age was negatively associated both with TSH (r=-0.05, p<0.0001; **Figure 5**) and fT4 levels (r=-0.04, p<0.01), also after controlling for BMI, gender, smoking habit, %FM and FFM (TSH: r=-0.06, p<0.0001; fT4: r=-0.05, p<0.01).

**Figure 6:**
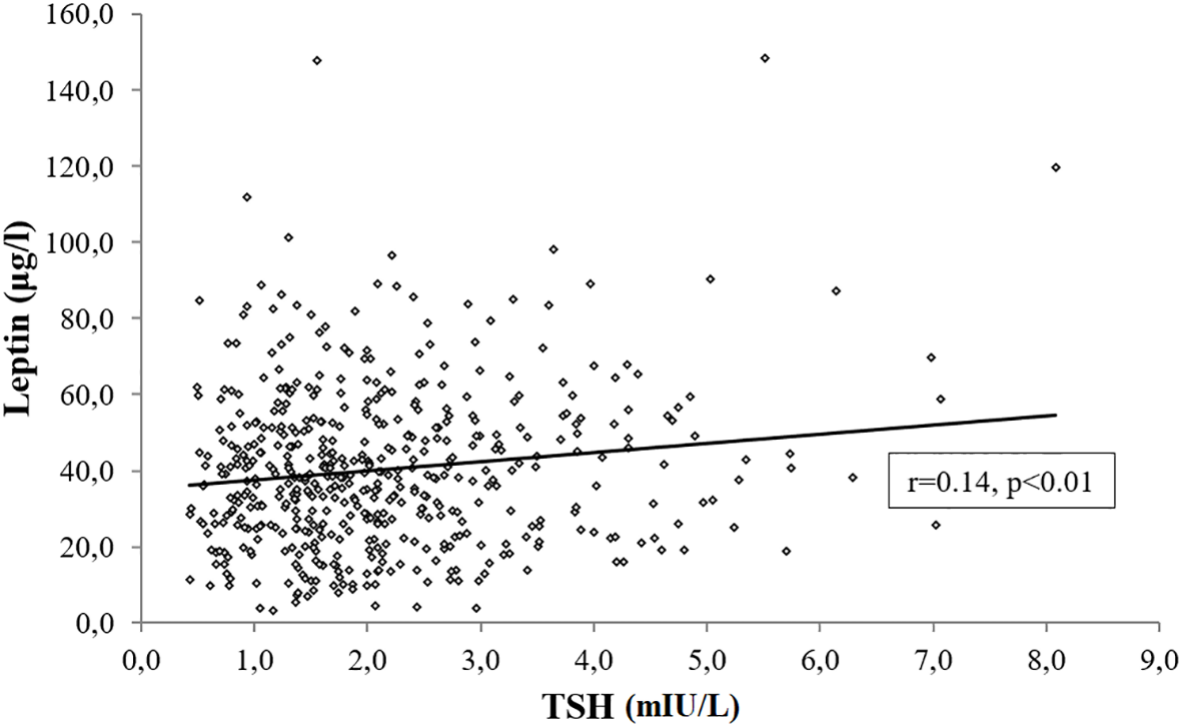
Bivariate correlation analysis between TSH and leptin levels in 502 subjects.

In a subset of 502 subjects with age, gender and BMI distribution comparable to the study cohort (Age, 48.6 ± 14.8 years; Sex, M/F:133/369; BMI, 43.6 ± 7.4 kg/m^2^), we measured leptin levels and documented its positive association with TSH (r=0.14 p<0.01) (**Figure 6**), which was unchanged after controlling for BMI, age, gender and smoking habit (r=0.11, p=0.01).

Multivariable linear regression models were built to identify the independent predictors of TSH and fT4 levels in the population as a whole. Due to the overlap between BMI and measures of body composition, these latter were used for the regression model based on the higher significance achieved at the bivariate correlation analysis. The model achieving the highest coefficient of determination (R2) included age, gender, smoking habit and FM as independent variables (**Table 4**). Smoking habit emerged as the main independent predictor of TSH (β=-0.24, p<0.0001) and fT4 (β=-0.25, p<0.0001) levels. Other independent predictors included age (TSH: β=-0.06, p<0.0001, fT4: β=-0.04, p<0.0001), FM (TSH: β=0.09, p<0.0001) and female gender (TSH: β=0.03, p<0.05).

**Table 4:** Multivariable linear regression analysis showing independent predictors for TSH and fT4 levels.

	Dependent variables			
	TSH (R^2^= 0.076)		fT4 (R^2 ^= 0.062)	
ModelIndependent variables	Beta	p-value	Beta	p-value
Smoking habit*	**-0.24**	**<0.0001**	**-0.25**	**<0.0001**
FM	**0.09**	**<0.0001**	-0.005	0.71
Age	**-0.06**	**<0.0001**	**-0.04**	**0.006**
Gender^§^	**0.03**	**0.044**	0.007	0.63

Significant associations are shown in bold characters.

*Smoking habit: No = 0, Yes = 1; ^§^Gender: M = 0, F = 1.

FM, fat mass.

Considering the impact of smoking, we further analyzed only non-smokers and documented that %FM (β=0.08, p<0.0001), age (β=-0.05, p<0.001) and female gender (β=0.06, p<0.01) were independent predictors of TSH, while age was the only predictor of fT4 levels (β=-0.04, p<0.05).

We lastly tested the subset of patients having the leptin levels measured and found that leptin (β=0.11, p<0.05) and smoking habit (β=-0.30, p<0.0001) were independent predictors of TSH levels (**Table 5**), while fT4 was predicted by age (β=0.10, p<0.05) and smoking status (β=-0.19, p<0.0001) (**Table 5**). In this subgroup, an analysis restricted to non-smokers showed that leptin was the only independent predictor of TSH levels (β=0.17, p<0.01).

**Table 5:** Multivariable linear regression analysis showing independent predictors for TSH and fT4 levels in the subgroup of patients with leptin measured.

	Dependent variables			
	TSH (R^2 ^= 0.110)		fT4 (R^2 ^ = 0.046)	
ModelIndependent variables	Beta	p-value	Beta	p-value
Smoking habit*	**-0.30**	**<0.0001**	**-0.19**	**<0.0001**
FM	0.06	0.19	-0.05	0.29
Age	-0.006	0.88	**0.10**	**0.03**
Gender^§^	-0.04	0.46	-0.02	0.73
Leptin	**0.11**	**0.02**	0.006	0.90

Significant associations are shown in bold characters.

*Smoking habit: No = 0, Yes = 1; §Gender: M = 0, F = 1.

BMI, body mass index; FFM, fat-free mass; FM, fat mass.

## Discussion

The present study evaluated profiles and metabolic correlates of TSH and fT4 in the largest cohort of people with obesity spanning a very wide range of BMIs. Current data show an upward trend of TSH in females and a downward trend of fT4 levels in males across incremental BMI classes. A declining trend for TSH and fT4 was noticed with increasing age, and both correlated with metabolic variables. Smoking was a strongly associated with circulating TSH and fT4 levels. In non-smokers, %FM, age and gender were significant predictors of TSH. In a patient subset undergoing leptin measurement, leptin and %FM emerged as the strongest predictors of TSH levels.

Obesity is associated with changes in the secretion pattern of several hormones (38) and causes slight increments in TSH levels even above the TSH normalcy threshold devoid of underlying thyroid dysfunction (3, 4, 11–13, 18, 33, 34). A global analysis of the determinants of thyroid function parameters in this condition remains elusive due to generally limited study samples, underrepresentation of severe degrees of obesity, exclusion of subtle covariates such as age, smoking status, gender and leptin levels, as well as inclusion of individuals with thyroid disease.

In a large group of subjects with obesity apparently free of thyroid dysfunction, mean TSH levels were 2.08 mIU/L and the reference range was 0.58-5.07 mIU/L (2.5–97.5 centiles). Compared to general population data, such figures are clearly shifted to higher values. In the NHANES III study of 13,344 people considered free of thyroid disorders, median TSH levels were 1.39 mIU/L with a reference range of 0.45–4.12 mIU/L, and a relatively long tail towards higher values (39). Likewise, a German population study of 1,488 subjects without ultrasonographic evidence of thyroid disease obtained median TSH levels of 0.76 mIU/L and a reference interval of 0.25–2.12 mIU/L (40). Also, two Danish studies performed in areas with mild-moderate iodine deficiency reported reference TSH intervals of 0.40–3.6 mIU/L (41) and 0.58-4.07 mIU/L (42), while a study in 58,684 disease-free subjects from mainland China proved that ethnicity can influence TSH, with reference interval being 0.74-7.04 mIU/L and a long tail toward high TSH values (43). It is recognized that more sensitive TSH assays, more accurate thyroid antibody tests, and a more accurate selection of the reference population allowed a progressive reduction of the upper TSH reference limit over the last decade (44). This circumstance raised a debate on the opportunity to rank the upper normal TSH at 2.5 mIU/L (44–47), which would lead to classify 28% of our patients as TSH-abnormal against the 5.5% rate noticed here. The peculiarity of studying patients with extreme obesity also allowed us to realize that the direct correlation between TSH and BMI at high BMIs is not linear, i.e. above a very high BMI threshold the levels of TSH does not seem to increase further.

With regards to metabolic determinants, both TSH and fT4 levels were correlated with differing metabolic variables, yet an impact of body composition, insulin and total cholesterol levels appeared only significant for TSH. This confirms evidence obtained in the euthyroid general population, suggesting that TSH and fT4 concentrations are associated with divergent metabolic markers and the combined use of TSH and fT4 is a more convenient approach to evaluate the association between thyroid function and metabolic variables (48). Accumulation of fat mass is an intrinsic determinant of changes in thyroid hormones homeostasis (6, 8, 12, 16, 20). It has been suggested that TSH elevation could reflect an adaptive response of the thyrotropic axis to increase energy expenditure, thus reducing weight excess (34). However, if the increase in TSH levels was the primary event of this response, an increase in serum thyroid hormones would also be expected. On the contrary, our results showed normal fT4 levels in the vast majority of our cases. To explain this finding, it should be considered that the thyroxine turnover rate is proportional to body size. Thus, an increased rate of thyroid hormone disposal resulting from a large body size could potentially constitute the causative event promoting the activation of the thyrotropic axis aimed at maintaining serum thyroid hormones within the normal range (34). In addition, some studies reported a moderate increase in total T3 or fT3 in patients with obesity (17–19). Progressive central fat accumulation has been found to be associated with an increase in TSH and fT3 levels irrespective of insulin sensitivity and metabolic variables. This finding could suggest a high conversion of T4 in T3 caused by an increase in deiodinase activity as a compensatory mechanism for fat accumulation to improve energy expenditure (18).

In this cohort, women had higher TSH levels, as well as higher adiposity and leptin levels than men. Literature data show conflicting results on the relation between TSH and gender, and most studies reported overlapping TSH levels between genders (43, 49–52), while others described either lower (53) or higher TSH levels (54) in females compared to males. The mechanism underlying this gender-based difference in TSH remains unclear. Animal studies provided controversial results, since estradiol administration increased the number of pituitary TRH binding sites and reversed the inhibitory thyroxine effect on TSH response to TRH (55), but caused no change in pituitary TSH content and serum TSH levels in euthyroid and untreated hypothyroid rats (56). Because gender-dependent divergences in fat mass and leptin levels were seen and both associate positively with TSH levels, we hypothesize the potential intervention of leptin, which is recognized to be gender-dependent (57) and is able to stimulate the HPT axis and TSH secretion (58). Hyper-leptinaemia could explain the high TSH levels seen in obesity, while estradiol stimulation of leptin (59) could represent the biological basis for gender differences of TSH levels in this cohort.

Smoking status was an independent predictor of low TSH and fT4 levels. Smoking status is known to influence thyroid function parameters (60, 61), body weight (62) and the association between TSH and BMI (10, 14, 32, 60, 63, 64). Potential mediators of the effects of smoking on thyroid function involve: 1) the sympathetic stimulation of thyroid hormone synthesis and release (64); 2) the inhibition of sodium-iodide symporter mediated by thiocyanate, a cyanide-transformation product (65); 3) increased type 2 deiodinase activity (66). Our study suggests, however, that the influence of smoking on TSH does not interfere on TSH relationship with body weight. We thus hypothesize that even in obesity smoking promotes non-physiological changes in thyroid function, with potential metabolic consequences that remain to be investigated.

With regards to the negative relationship herein observed between thyroid function parameters and age, our finding contrasts with previous longitudinal studies showing that aging is associated with increasing serum TSH concentrations devoid of changes in fT4 levels (39, 67). While this latter evidence suggests the involvement of age-dependent alterations in TSH set-point and/or lower pituitary sensitivity to thyroid hormones and/or or reduced TSH bioactivity rather than occult thyroid disease (68), our findings agree with previous findings of declining TSH levels with aging documented in the general population (69–72). Some argued that, in case of insufficient or borderline-sufficient iodine intake, a decrease in serum TSH throughout life could reflect a late compensatory condition of thyroid autonomy (70). Others suggested that age modifies the pituitary set-point, based on the evidence that TSH secretion decreases in response to TRH in aging individuals and that TSH response to decreased thyroid hormone concentrations is impaired in older adults, thus implying some form of age-induced thyrotropic cell insensitivity (71, 72). Alternatively, based on aging effects on body composition and fat compartmentalization from subcutaneous to visceral sites (73), serum leptin levels and/or its pattern of secretion may change with age (57, 74–76) and hamper the central control of TRH and TSH secretion. In addition, some authors hypothesized a condition of leptin resistance associated with an increase of systemic inflammation that could contribute to alter the hypothalamus set-point (77). Finally, it is worth mentioning that an impairment of peripheral 5’-deiodinase and an increase in reverse 3,5,3’-triiodothyronine due to non-thyroidal illness are argued to intervene in the relationship between thyroid function and age (69).

Our study illustrates adiposity and metabolic determinants of TSH levels in a large cohort of patients with obesity. Given its retrospective nature, this study has a number of limitations that include the lack of fT3 measurement and the inability to assess mechanisms responsible for observed associations. Iodine status remains a major determinant of reference intervals for TSH, thus it remains to be established whether TSH elevation in obesity may result from impaired iodine intake or other nutritional causes since iodine deficiency, more than adequate or excess iodine, is a risk factor for hyperthyrotropinemia (78, 79).

In conclusion, our study in a large population with obesity established that TSH levels show an upward trend with increasing degree of obesity in females and leptin levels emerged as the main metabolic determinant of thyroid function parameters in obesity.

## Data availability statement

The original contributions presented in the study are included in the article/supplementary material. Further inquiries can be directed to the corresponding author.

## Ethics statement

The studies involving human participants were reviewed and approved by Ethics Committee of Istituto Auxologico Italiano. The patients/participants provided their written informed consent to participate in this study.

## Author contributions

CM and PM designed the study and draft the manuscript; CM, PM, LP, and BB interpreted the results and contributed to the discussion; CM, SM, and TC collected and analyzed data; GA, MS, and BB contributed to analyze data and reviewed the manuscript; GA and PM reviewed and edited the manuscript. All authors contributed to the article and approved the submitted version.

## Funding

Research founded by the Italian Ministry of Health (18C101_2011).

## Conflict of interest

The authors declare that the research was conducted in the absence of any commercial or financial relationships that could be construed as a potential conflict of interest.

## Publisher’s note

All claims expressed in this article are solely those of the authors and do not necessarily represent those of their affiliated organizations, or those of the publisher, the editors and the reviewers. Any product that may be evaluated in this article, or claim that may be made by its manufacturer, is not guaranteed or endorsed by the publisher.
